# Association of antibiotic-consumption patterns with the prevalence of hematological malignancies in European countries

**DOI:** 10.1038/s41598-022-11569-y

**Published:** 2022-05-12

**Authors:** Gábor Ternák, Károly Berényi, Balázs Németh, Ágnes Szenczi, Gergely Márovics, István Kiss

**Affiliations:** 1 Institute of Migration Health, University of Pécs, Medical School, Szigeti st. 12., Pecs, 7624 Hungary; 2 Department of Public Health Medicine, University of Pécs, Medical School, Szigeti st. 12., Pecs, 7624 Hungary

**Keywords:** Medical research, Haematological diseases, Haematological cancer

## Abstract

Hematological malignancies are considered the fifth most common cancer in the world. Several risk factors and probable etiological agents have been suspected in the pathomechanism of those malignancies as infections, chemicals, irradiation, etc., and recently, the contribution of the altered gut flora, dysbiosis, was identified also as a possible additional factor to the existing ones. Host, and external factors, like antibiotics, which were identified as a major disruptor of the "normal" gut flora, influence the composition of the microbiome. Considering the several-fold differences in antibiotic consumption patterns and the incidence of hematological malignancies in European countries, the hypothesis was raised that the dominant consumption of certain antibiotic classes might influence the incidence of different hematological malignancies through the modification of gut flora. Comparisons were performed between the average antibiotic consumption databases reported yearly by ECDC (2009–2019) and the incidence rate of Hodgkin lymphoma (HL), non-Hodgkin lymphoma (NHL), multiple myeloma (MM), and leukemia (LEU) estimated for 2020 in 30 European countries. Applying Spearman calculations, significant positive correlation has been found between the incidence of HL and tetracycline (J01A) consumption (*r* = *0.399, p* = *0.029),* NHL and narrow spectrum, beta-lactamase resistant penicillin (J01CF) (*r* = *0.580, p* = *0.001),* MM and tetracycline (*r* = *0.492, p* = *0.006),* penicillin (J01C) (*r* = *0.366, p* = *0.047),* narrow spectrum, beta-lactamase resistant penicillin (J01CF) (*r* = *0.574, p* = *0.001*), while strong, significant negative correlation has been recorded between NHL and cephalosporin (*r* = *− 0.460, p* = *0.011),* and quinolone (*r* = *− 0.380, p* = *0.038*)*.* The incidence of LEU did not show any positive or negative association with any antibiotic classes using Spearman calculation. Multivariate ordinal logistic regression (OR) indicated increased risk between HL and the total consumption of systemic antibiotics (J01 *p: 0.038*), and tetracyclin (J01A *p: 0.002*). Similarly, increased risk has been detected between the MM and tetracyclin (J01A *p: 0.02*), and narrow spectrum, beta-lactamase resistant penicillin (J01CF *p: 0.042*) and decreased risk between cephalosporin and MM (J01D *p:0.022*). LEU showed increased risk with the consumption of macrolides (*p: 0.047*).

## Introduction

Hematologic malignancies (HMs) are the fifth most common cancer group in economically developed countries of the world and they originate from the uncontrolled proliferation of hematopoietic and lymphoid cells. Recent publications have indicated the possible role of altered microbiome (dysbiosis) as a causative factor in oncogenesis, which might develop as the effect of external factors, like antibiotics. The authors identified some classes of antibiotics, which might promote (tetracycline, penicillin) or inhibit (cephalosporin, quinolone) the development of certain hematological malignancies probably through generating dysbiosis, as their incidence showed significant positive or negative associations with the antibiotic consumption patterns in 30 European countries.

Cancer cases increased by 28% between 2006 and 2016, but disparity has been observed between different countries with low and high Sociodemographic Index (SDI), and the smallest increase was seen in high SDI countries, but despite the rapidly increasing cancer burden in lower SDI countries, the probability of developing cancer (age-standardized rates) are still higher in countries of higher SDI. Notable exceptions are cancers with infectious etiologies like cervical (Human Papilloma Virus /HPV/), liver (Hepatitis B Virus /HBV/, Hepatitis C Virus/HCV/), and stomach cancer (*H. pylori*)^1^.

Recently, worldwide, an estimated 19.3 million new cancer cases (18.1 million excluding nonmelanoma skin cancer) and almost 10.0 million cancer deaths (9.9 million excluding nonmelanoma skin cancer) occurred in 2020^2^. Cancers, due to infectious agents were estimated at 2.2 million in 2018, but exact numbers could not be ascertained due to scarcity of local data, especially in low-SDI countries^3^. Hematologic malignancies (HMs) are the fifth most common cancer group in economically developed countries of the world and they originate from the uncontrolled proliferation of hematopoietic and lymphoid cells. These biologically and clinically heterogeneous groups account for 6.5% of all malignancies around the world, including approximately 9.0% in the United States and Europe^4–6^. They are traditionally categorized by site according to whether cancer is first detected in the blood (leukemias), lymph nodes (lymphomas—Hodgkin and non-Hodgkin), or bone (myelomas)^7^.

*Hodgkin lymphoma (HL)*, is called Hodgkin’s disease (HD), considered as one of many types of lymphoma and the most common type of HD is the “classic” (CHD, 95%) form containing abnormal lymphocytes, known as Reed-Sternberg cells with an estimated annual incidence of 80,000 cases worldwide^8,9^.

*Non-Hodgkin lymphoma (NHL)*: The development of NHL begins when healthy B cells, T cells, or NK cells in the lymphatic system change and grow out of control, which may form a tumor^10,11^.

*Multiple myeloma (MM)* Multiple myeloma is a cancer of plasma cells. In 2016, there were about 130,000 cases of myeloma, translating to an age-standardized incidence rate of 2.1 per 100,000 persons. Multiple myeloma caused 98,437 deaths globally, with an age-standardized incidence ratio of 1.5 per 100,000 persons. That means from 1990 to 2016, incident cases of MM increased by 126% globally and deaths increased 94%^12^.

*Leukemia (LEU)* is the cancer of the body's blood-forming cells**,** including the bone marrow and the lymphatic system. Several types of leukemia exist. They might develop in children or adults and they can appear as acute, or chronic diseases. Leukemia represents the 11th and 10th most frequent cause of cancer occurrence and death worldwide^13^. Risk factors and/or putative causes for hematological malignancies are gender, age, exposure to chemicals (benzene), radiation, congenital syndromes (Fanconi, Dawn, Bloom syndrome, etc.), viral infections (Epstein-Barr virus, Human T-cell Leukemia Virus /HTLV/, hepatitis-C, HIV/AIDS, etc.), bacterial infections (*H. pylori* in MALT lymphoma)^14–22^.

The human microbiome has several beneficial effects in terms of maintaining appropriate human health, but its alteration has been implicated in the development of many illnesses. and gut microbiota dysbiosis—imbalances in the composition and function of these intestinal microbes—is associated with diseases ranging from localized gastroenterology disorders to neurologic, respiratory, metabolic, hepatic, and cardiovascular illnesses. Gut microbial species are being explored in the field of oncology also. Of specific interest is the capacity of some commensal bacteria to modulate the tumor microenvironment and anticancer therapies^23^. In one of our previous works, associations have been observed between antibiotic consumption patterns and the incidence of major cancers in European countries^24^. Similarly, repeated antibiotic consumption is associated with cancer prevalence, probably acting through the modification of the gut microbiome^25^. Publications indicate the possible role of the different intestinal microbiome in the development of hematological malignancies and even anticancer treatment. The microbiome can influence hematologic malignancies in several ways, including directly through metabolites and toxins, or indirectly via the innate and adaptive immune system^26–28^.

### Concept/hypothesis

Antibiotic consumption patterns in European countries are extremely different. The most preferred antibiotics used in certain countries are narrow-spectrum penicillin and tetracycline, while in others; broad-spectrum antibiotics are most frequently consumed. The calculated average ratio of broad/narrow-spectrum antibiotics for the years of 2010–2019 (10 years, expressed in Defined Daily Dose/1000 inhabitants/Day /DID/) is the highest in Greece (321.94) and the lowest is in Norway (0.19). Based on this 1694.42 fold difference it could be suspected that those very different antibiotic consumption patterns might influence the composition of the gut flora differently and hence, the altered gut flora (dysbiosis) might promote, or inhibit the development of certain ailments. The incidence of different hematological cancers (HL NHL, MM, LEU, 29–32) estimated for 2020 shows considerable differences between European countries. The highest incidence rate for HD is in Cyprus (4.69), the lowest is in Romania (1.3). NHL incidence is highest in Slovenia (28.1), the lowest is in Bulgaria (8.7). The MM incidence is highest in Iceland (11.7), the lowest is in Bulgaria (2.2). The incidence of LEU is highest in Belgium (21) and the lowest is in Bulgaria (7.5). The difference between the highest and lowest incidence rate of the above hematological malignancies is approximately three to fivefold^29–32^.

It can be hypothesized that different classes of antibiotics, producing different modifications on the gut flora, might promote or inhibit the development of different hematological malignancies and this activity could be attached to different antibiotic classes. We have hypothesized also that if antibiotics, through different putative mechanisms, published in the scientific literature, could influence the hematological oncogenesis, those antibiotic consumption patterns might be reflected in the incidence of different hematological malignancies in the different countries included in the study.

## Materials and methods

**Figure 1:**
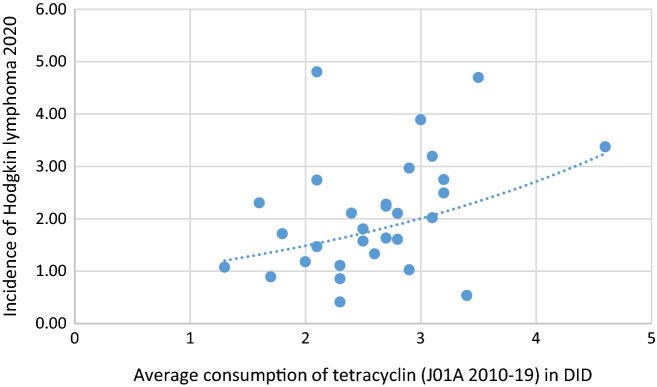
A significant positive association has been found between tetracycline consumption and the incidence of HL (2020).

**Figure 2:**
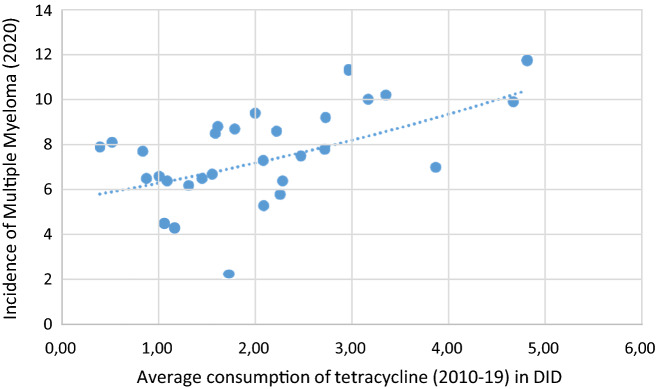
A significant positive association was seen between tetracycline consumption and the incidence of MM (2020).

**Figure 3:**
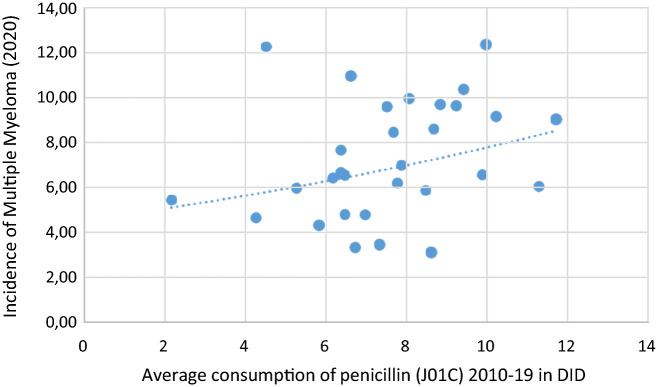
A significant positive association was found between penicillin consumption and the incidence of MM (2020).

**Figure 4:**
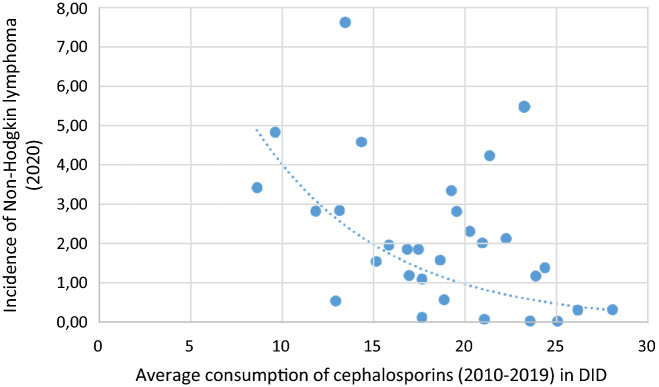
A significant negative association was found between cephalosporin consumption and the incidence of NHL (2020).

Databases were calculated from publicly available antibiotic consumption figures (ECDC yearly reports) for 2009–2019^33^ and the incidence of hematological malignancies (HD, NHD, MM, LEU) estimated for 2020 and featured in the European Cancer Information System (ECIS) for 30 European countries. Average yearly consumption of total systemic antibiotics (ATC classification J01) expressed in Defined Daily Dose/1000 Inhabitants/Day (DID) was calculated similarly with major antibiotic classes at ATC level 3 and 4 as tetracycline (J01A), penicillin (J01C), broad-spectrum, beta-lactamase sensitive penicillin (J01CA), narrow spectrum, beta-lactamase sensitive penicillin (J01CE), narrow spectrum, beta-lactamase resistant penicillin (J01CF), broad-spectrum, beta-lactamase resistant combination penicillin (J01CR), cephalosporin (J01D), macrolide and lincosamides, streptogramins (J01F), a quinolone (J01M). The average ratio of broad/narrow-spectrum (B/N) antibiotic consumption/countries have been calculated also. Antibiotic consumption data and the incidence of hematological malignancies were recorded by countries and featured in a spreadsheet (Table 1). Diagrams for demonstrating positive and negative associations between certain hematological malignancies and antibiotic consumption data were created (Figs. 1, 2, 3, 4).

**Table 1:** Comparison of average antibiotic consumption (2010–2019) expressed in Defined Daily Dose/ 1000 inhabitants/ day (DID), and the incidence of hematologic malignancies estimated for 2020.

Countries	Average. total antibiotic consumpti on 2010–2019 in DID	Antibiotic classes Average consumption (2010–2019) of tetracycline (J01A). penicillin (J01C). beta-lactamase sensitive broad-spectrum penicillin (J01CA). beta-lactamase sensitive. narrow-spectrum penicillin (J01CE). beta-lactamase resistant. narrow-spectrum penicillin (J01CF). broad- spectrum. beta-lactamase resistant combination penicillin (J01CR). cephalosporin (J01D). macrolide (and lincosamides. streptogramines). quinolone (J01F). quinolone (J01M). ratio of broad-/narrow spectrum antibiotics *expressed in Defined Daily Dose/ 1000 inhabitants/ day* *(DID)*										Incidence of hematological malignancies 2020 (100,000/cases)			
Country	J01	J01A	J01C	J01CA	J01CE	J01CF	J01CR	J01D	J01F	J01M	B/N	*HL*	*NHL*	*MM*	*LEU*
Austria	**12**	**0.89**	**4.79**	**0.79**	**0.79**	**0.01**	**3.21**	**1.55**	**2.98**	**1.22**	**6.71**	***1.7***	***15.2***	***6.5***	***14***
Belgium	**22.25**	**2.02**	**10.33**	**4.94**	**0.03**	**0.26**	**5.1**	**1.4**	**3.4**	**2.23**	**85.39**	***3.1***	***24.4***	***9.4***	***21***
Bulgaria	**17.87**	**1.72**	**5.43**	**3.13**	**0.19**	**0**	**2.11**	**3.42**	**3.65**	**2.61**	**27.34**	***1.8***	***8.7***	***2.2***	***7.5***
Croatia	**17.32**	**1.11**	**7.64**	**1.97**	**0.66**	**0**	**5**	**2.84**	**2.88**	**1.44**	**8.21**	***2.3***	***13.2***	***6.4***	***12.1***
Cyprus*	**26.64**	**3.38**	**9.14**	**2.43**	**0.08**	**0.02**	**6.62**	**5.47**	**2.92**	**4.75**	**32.43**	***4.6***	***23.2***	***10.2***	***18.9***
Czechia	**16.64**	**2.11**	**5.96**	**1.16**	**1.85**	**0.05**	**2.88**	**1.86**	**3.67**	**0.95**	**4.28**	***2.4***	***17.5***	***5.3***	***14.2***
Denmark	**15.1**	**1.63**	**9.61**	**3.19**	**4.28**	**1.39**	**0.74**	**0.03**	**1.91**	**0.48**	**0.55**	***2.7***	***25.1***	***8.8***	***15.4***
Estonia	**10.25**	**1.58**	**3.31**	**1.72**	**0.19**	**0**	**1.4**	**1.1**	**2.36**	**0.83**	**12.38**	***2.5***	***17.7***	***6.7***	***14.7***
Finland	**15.73**	**3.89**	**4.78**	**2.65**	**1.24**	**0.04**	**0.85**	**2.13**	**1.13**	**0.76**	**0.59**	***3***	***22.3***	***7***	***11.5***
France	**23.54**	**3.19**	**12.37**	**7.34**	**0.17**	**0.23**	**4.64**	**2.03**	**3.27**	**1.63**	**36.71**	***3.1***	***21***	***10***	***16.8***
Germany	**12.97**	**2.11**	**3.43**	**2.24**	**0.75**	**0.01**	**0.44**	**2.82**	**2.32**	**1.25**	**5.67**	***2.8***	***19.6***	***7.3***	***14.8***
Greece*	**31.19**	**2.49**	**9.56**	**4.42**	**0.08**	**0**	**5.07**	**7.61**	**7.5**	**2.63**	**321.94**	***3.2***	***13.4***	***7.5***	***15.4***
Hungary	**13.62**	**1.18**	**4.63**	**0.88**	**0.26**	**0**	**3.49**	**1.96**	**2.93**	**2.22**	**42.74**	***2***	***15.9***	***4.3***	***13.6***
Iceland*	**19.03**	**4.81**	**9.03**	**3.23**	**2.08**	**1.04**	**2.69**	**0.58**	**1.61**	**0.91**	**1.5**	***2.1***	***18.9***	***11.7***	***11.3***
Ireland	**19.83**	**2.75**	**9.56**	**2.96**	**1.06**	**1.4**	**4.15**	**1.18**	**4.12**	**0.83**	**4.8**	***3.2***	***23.9***	***9.2***	***16.3***
Italy	**21.7**	**0.54**	**9.97**	**2.61**	**0**	**0.01**	**7.35**	**2.32**	**4.49**	**3.13**	**165.5**	***3.4***	***20.3***	***8.1***	***13.4***
Latvia	**11.14**	**2.28**	**4.29**	**2.89**	**0.05**	**0**	**1.35**	**0.55**	**1.72**	**0.99**	**12.46**	***2.7***	***13***	***5.8***	***14.3***
Lithuania*	**13.9**	**1.47**	**6.53**	**4.9**	**0.23**	**0**	**1.41**	**1.2**	**1.99**	**0.95**	**9.29**	***2.1***	***17***	***6.5***	***18.3***
Luxembourg	**21.89**	**1.81**	**8.6**	**3.13**	**0.02**	**0.18**	**5.29**	**3.35**	**3.9**	**2.5**	**48**	***2.5***	***19.3***	***8.7***	***15.2***
Malta	**19.26**	**1.33**	**6.41**	**0.49**	**0.1**	**0.06**	**5.77**	**4.24**	**3.92**	**2.3**	**100.25**	***2.6***	***21.4***	***6.2***	***9.7***
Netherlands	**9.44**	**2.24**	**3.07**	**1.31**	**0.26**	**0.44**	**1.06**	**0.04**	**1.42**	**0.77**	**8.82**	***2.7***	***23.6***	***8.6***	***13.6***
Norway	**15.16**	**2,97**	**6.03**	**2.12**	**3.27**	**0.63**	**0.01**	**0.09**	**1.42**	**0.45**	**0.19**	***2.9***	***21.1***	***11.3***	***15.6***
Poland	**20.9**	**2.3**	**6.66**	**3.42**	**0.24**	**0.01**	**2.98**	**2.82**	**4.18**	**1.32**	**28.22**	***1.6***	***11.9***	***6.4***	***12.6***
Portugal	**17.65**	**0.86**	**8.44**	**1.7**	**0.02**	**0.53**	**6.19**	**1.59**	**2.99**	**2.14**	**36.51**	***2.3***	***18.7***	***7.7***	***13.7***
Romania	**25.76**	**1.08**	**12.23**	**4.63**	**0.8**	**0.66**	**6.14**	**4.83**	**2.91**	**3.33**	**12.11**	***1.3***	***9.7***	***4.5***	***9.9***
Slovakia	**19.92**	**1.61**	**5.86**	**1**	**1.21**	**0**	**3.65**	**4.58**	**5.3**	**1.94**	**9.52**	***2.8***	***14.4***	***8.5***	***16.1***
Slovenia	**11.72**	**0.41**	**6.97**	**2.27**	**1.68**	**0.16**	**2.86**	**0.33**	**1.8**	**1.1**	**2.94**	***2.3***	***28.1***	***7.9***	***15***
Spain	**19.78**	**1.03**	**10.94**	**4.34**	**0.09**	**0.21**	**6.32**	**1.86**	**2.43**	**2.53**	**54.65**	***2.9***	***16.9***	***6.6***	***12.3***
Sweden	**12.25**	**2.74**	**6.18**	**1.07**	**3.38**	**1.5**	**0.22**	**0.14**	**0.59**	**0.68**	**0.2**	***2.1***	***17.7***	***7.8***	***13***
UK	**17.18**	**4.7**	**6.55**	**3.51**	**0.82**	**1.44**	**0.78**	**0.32**	**2.93**	**0.45**	**1.44**	***3.5***	***26.2***	***9.9***	***17***
**HL**															
Spearman *r*	0.321	0.399	0.267	0.22	− 0.227	0.192	0.125	0.003	0.146	− 0.004	0.137				
Spearman *p*	0.84	***0.029***	0.154	0.243	0.227	0.311	0.51	0.987	0.441	0.985	0.469				
OR	*1.169*	*3.141*	1.163	1.48	0.955	1.238	1.141	0.688	1.935	1.485	1.017				
OR CI95%	*1.017–1.375*	*1.316–9.336*	0.831–1.663	0.898–2.643	0.386–2.374	0.163–8.787	0.789–1.671	0.272–1.701	0.780–5.072	0.392–6.305	1.001–1.042				
*p*	***0.038***	***0.02***	0.385	0.144	0.919	0.827	0.483	0.414	0.158	0.563	0.103				
**NHL**															
Spearman *r*	− 0.087	0.257	0.161	0.023	0.163	0.58	− 0.154	− 0.46	− 0.225	− 0.38	− 0.27				
Spearman *p*	0.649	0.171	0.394	0.902	0.389	***0.001***	0.417	***0.011***	0.232	***0.038***	0.149				
OR	0.949	2.023	1.012	0.976	1.027	4.637	0.911	0.484	0.97	1.792	0.994				
OR CI95%	0.825–1.085	0.939–4.983	0.714–1.424	0.608–1.547	0.369–2.864	0.696–43.155	0.621–1.322	0.176–1.173	0.404–2.380	0.452–7.189	0.980–1.005				
*p*	0.438	0.09	0.944	0.918	0.959	0.13	0.625	0.123	0.945	0.394	0.32				
**MM**															
Spearman *r*	0.223	0.492	0.366	0.231	0.12	0.574	− 0.07	− 0.34	− 0.126	− 0.272	− 0.222				
Spearman *p*	0.236	***0.006***	***0.047***	0.22	0.526	***0.001***	0.715	*0.063*	0.506	0.146	0.238				
OR	1.06	*6.446*	1.383	1.492	1.031	*11.805*	1.04	*0.292*	2.141	2.322	1				
OR CI95%	0.936–1.213	*2.209–25.494*	0.930–2.138	0.893–2.665	0.325–3.257	*1.430–196.582*	0.693–1.564	*0.088–0.777*	0.832–6.603	0.611–10.379	0.990–1.009				
*p*	0.367	***0.002***	0.118	0.139	0.957	***0.042***	0.849	***0.022***	0.136	0.225	0.925				
**LEU**															
Spearman *r*	0.112	0.267	0.174	0.258	− 0.068	0.088	− 0.068	− 0.2	− 0.126	− 0.161	− 0.036				
Spearman *p*	0.557	0.154	0.358	0.168	0.719	0.643	0.722	0.278	0.506	0.395	0.849				
OR	1.044	1.688	1.033	1.46	1.02	1.503	0.857	0.561	*2.692*	0.9	1.002				
OR CI95%	0.917–1.192	0.748–4.233	0.732–1.450	0.892–2.523	0.402–2.577	0.222–10.970	0.586–1.239	0.207–1.370	*1.072–7.942*	0.183–3.789	0.990–1.016				
*p*	0.508	0.227	0.849	0.141	0.965	0.67	0.414	0.219	***0.047***	0.89	0.686				

Spearman correlation indicated (bold, *italics*, underlined) significant positive associations between HL and tetracycline (J01A), NHL and narrow spectrum, beta-lactamase resistant penicillin (J01CF), MM and tetracycline (J01A), and penicillin (J01C) particularly with the narrow spectrum, beta-lactamase resistant penicillin (J01CF). Negative significance was found (bold, *italics*) between NHL and cephalosporin (J01D) and quinolone (J01M). The tendency for a positive correlation between HD and the total consumption of systemic antibiotics (J01, bold) was observed also. A tendency for negative correlation has been detected between MM and cephalosporin (J01D, bold). Multivariate ordinal logistic regression (OR) indicated increased risk between HL and the total consumption of systemic antibiotics (J01 *p: 0.038*), and tetracyclin (J01A *p: 0.002*). Similarly, increased risk has been detected between the MM and tetracyclin (J01A *p: 0.02*), and narrow spectrum, beta-lactamase resistant penicillin (J01CF *p: 0.042*) and decreased risk between cephalosporin and MM (J01D *p: 0.022*). LEU showed increased risk with the consumption of macrolides (*p: 0.047*).

*Countries provided hospital and OPD consumption of antibiotics together.

### Statistics

Spearman correlation was applied to estimate the correlation between antibiotic consumption and the prevalence data of hematological malignancies. A significant correlation was considered when *p* values were ≤ 0.05. Non-significant correlation was estimated when the p values fall between 0.05 and 0.09. Positive (supportive) and negative (non-supportive) significant correlations were considered and evaluated. Statistical results were recorded and featured in the same table (Table 1).

The homogeneity and the normality of data has been estimated by using Levene and Kolmogorov–Smirnov tests. We have found that certain variables are not identical and do not follow normal distribution. Multivariate ordinal logistic regression (OR) was used to examine the interfering effects of antibiotic usage. Results of the analysis are presented in Table 1. The spreadsheet was formulated for comparing the rank order of countries (first ten positions) with the highest incidence of different hematological malignancies and the rank order of consumption of antibiotic classes showing positive (“enhancing”) and negative (“inhibiting”) correlation with the hematological malignancies in the same countries (Table 2.).

**Table 2:** Decreasing rank order of “enhancer” antibiotic consumption (J01A, J01CF, J01C) compared to the decreasing rank order of hematological malignancies (HL, NHL, MM, LEU) by countries (first ten positions).

Decreasing rank order of “enhancer” antibiotic consumption (tetracycline: J01A, penicillin group: (J01C), narrow spectrum, penicillinase-resistant penicillin :J01CF) in European countries (average consumption for 2010–2019 in DID), compared to the decreasing rank orders of the incidence of the major groups of hematological malignancies 2020 (HL, NHL, MM, LEU)															
Countries	J01A	Countries	*HL*	Countries	J01A	Countries	*NHL*	Countries	J01A	Countries	*MM*	Countries	J01A	Countries	*LEU*
Iceland	4.81	***Cyprus***	***4.6***	Iceland	4.81	Slovenia	28.1	***Iceland***	***4.81***	***Iceland***	***11.7***	***Iceland***	***4.81***	Belgium	21
***UK***	***4.7***	***UK***	***3.5***	***UK***	***4.7***	***UK***	***26.2***	***UK***	***4.7***	***Norway***	***11.3***	***UK***	***4.7***	***Cyprus***	***18.9***
***Finland***	***3.89***	Italy	3.4	***Finland***	***3.89***	Denmark	25.1	Finland	3.89	***Cyprus***	***10.2***	Finland	3.89	Lithuania	18.3
***Cyprus***	***3.38***	***Greece***	***3.2***	***Cyprus***	***3.38***	Belgium	24.4	***Cyprus***	***3.38***	***France***	***10***	***Cyprus***	***3.38***	***UK***	***17***
***France***	***3.19***	***Ireland***	***3.2***	France	3.19	***Ireland***	***23.9***	***France***	***3.19***	***UK***	***9.9***	***France***	***3.19***	***France***	***16.8***
***Norway***	***2.97***	Belgium	3.1	***Norway***	***2.97***	Netherlands	23.6	***Norway***	***2.97***	Belgium	9.4	***Norway***	***2.97***	***Ireland***	***16.3***
***Ireland***	***2.75***	***France***	***3.1***	***Ireland***	***2.75***	***Cyprus***	***23.2***	***Ireland***	***2.75***	***Ireland***	***9.2***	Ireland	2.75	Slovakia	*16.1*
Sweden	2.74	***Finland***	***3***	Sweden	2.74	***Finland***	***22.3***	Sweden	2.74	Denmark	*8.8*	Sweden	2.74	***Norway***	***15.6***
***Greece***	***2.49***	***Norway***	***2.9***	Greece	2.49	Malta	21.4	Greece	2.49	Luxembourg	*8.7*	***Greece***	***2.49***	Denmark	15.4
Poland	2.3	Spain	2.9	Poland	2.3	***Norway***	**21.1**	Poland	2.3	Netherlands	*8.6*	Poland	2.3	***Greece***	***15.4***

Countries	J01CF	Countries	*HL*	Countries	J01CF	Countries	*NHL*	Countries	J01CF	Countries	*MM*	Countries	J01CF	Countries	*LEU*
Sweden	1.5	Cyprus	4.6	Sweden	1.5	Slovenia	28.1	Sweden	1.5	***Iceland***	***11.7***	Sweden	1.5	***Belgium***	***21***
***UK***	***1.44***	***UK***	***3.5***	***UK***	***1.44***	***UK***	***26.2***	***UK***	***1.44***	***Norway***	***11.3***	***UK***	***1.44***	Cyprus	18.9
***Ireland***	***1.4***	Italy	3.4	***Ireland***	***1.4***	***Denmark***	***25.1***	***Ireland***	***1.4***	Cyprus	10.2	***Ireland***	***1.4***	Lithuania	18.3
Denmark	1.39	Greece	3.2	***Denmark***	***1.39***	***Belgium***	***24.4***	***Denmark***	***1.39***	France	10	***Denmark***	***1.39***	***UK***	***17***
Iceland	1.04	***Ireland***	***3.2***	Iceland	1.04	***Ireland***	***23.9***	***Iceland***	***1.04***	***UK***	***9.9***	Iceland	1.04	France	16.8
Romania	0.66	***Belgium***	***3.1***	Romania	0.66	***Netherlands***	***23.6***	Romania	0.66	***Belgium***	***9.4***	Romania	0.66	***Ireland***	***16.3***
***Norway***	***0.63***	France	3.1	***Norway***	***0.63***	Cyprus	23.2	***Norway***	***0.63***	***Ireland***	***9.2***	***Norway***	***0.63***	Slovakia	16.1
Portugal	0.53	Finland	3	Portugal	0.53	Finland	22.3	Portugal	0.53	***Denmark***	***8.8***	Portugal	0.53	***Norway***	***15.6***
Netherlands	0.44	***Norway***	***2.9***	***Netherlands***	***0.44***	Malta	21.4	***Netherlands***	***0.44***	Luxembourg	8.7	Netherlands	0.44	***Denmark***	***15.4***
***Belgium***	***0.26***	Spain	2.9	***Belgium***	***0.26***	***Norway***	***21.1***	***Belgium***	***0.26***	***Netherlands***	***8.6***	***Belgium***	***0.26***	Greece	15.4

Countries	J01C	Countries	*HL*	Countries	J01C	Countries	*NHL*	Countries	J01C	Countries	*MM*	Countries	J01C	Countries	*LEU*
***France***	***12.37***	***Cyprus***	***4.6***	France	12.37	Slovenia	28.1	***France***	***12.37***	***Iceland***	***11.7***	***France***	***12.37***	***Belgium***	***21***
Romania	12.23	UK	3.5	Romania	12.23	UK	26.2	Romania	12.23	Norway	11.3	Romania	12.23	***Cyprus***	***18.9***
***Spain***	***10.94***	***Italy***	***3.4***	Spain	10.94	***Denmark***	***25.1***	Spain	10.94	***Cyprus***	***10.2***	Spain	10.94	Lithuania	18.3
***Belgium***	***10.33***	***Greece***	***3.2***	***Belgium***	***10.33***	***Belgium***	***24.4***	***Belgium***	***10.33***	***France***	***10***	***Belgium***	***10.33***	UK	17
***Italy***	***9.97***	***Ireland***	***3.2***	Italy	9.97	***Ireland***	***23.9***	Italy	9.97	UK	9.9	Italy	9.97	***France***	***16.8***
Denmark	9.61	***Belgium***	***3.1***	***Denmark***	***9.61***	Netherlands	23.6	***Denmark***	***9.61***	***Belgium***	***9.4***	***Denmark***	***9.61***	***Ireland***	***16.3***
***Greece***	***9.56***	***France***	***3.1***	Greece	9.56	***Cyprus***	***23.2***	Greece	9.56	***Ireland***	***9.2***	Greece	9.56	Slovakia	16.1
***Ireland***	***9.56***	Finland	3	***Ireland***	***9.56***	Finland	22.3	***Ireland***	***9.56***	***Denmark***	***8.8***	***Ireland***	***9.56***	Norway	15.6
***Cyprus***	***9.14***	Norway	2.9	***Cyprus***	***9.14***	Malta	21.4	***Cyprus***	***9.14***	Luxembourg	8.7	***Cyprus***	***9.14***	***Denmark***	***15.4***
Iceland	9.03	***Spain***	***2.9***	Iceland	9.03	Norway	21.1	***Iceland***	***9.03***	Netherlands	8.6	Iceland	9.03	Greece	15.4

Increasing (inverse) rank order of the average consumption (2010–2019, in DID) of “protective” (inhibitory) antibiotics (cephalosporin: J01D, quinolone: J01M), compared to the reducing rank order of the groups of hematological malignancies (incidence of HL, NHL, MM, LEU 2020)															
Countries	J01D	Countries	*HL*	Countries	J01D	Countries	*NHL*	Countries	J01D	Countries	*MM*	Countries	J01D	Countries	*LEU*
Denmark	0.03	Cyprus	4.6	***Denmark***	***0.03***	***Slovenia***	***28.1***	***Denmark***	***0.03***	***Iceland***	***11.7***	***Denmark***	***0.03***	Belgium	21
Netherlands	0.04	***UK***	***3.5***	***Netherlands***	***0.04***	***UK***	***26.2***	***Netherlands***	***0.04***	***Norway***	***11.3***	Netherlands	0.04	Cyprus	18.9
***Norway***	***0.09***	Italy	3.4	***Norway***	***0.09***	***Denmark***	***25.1***	***Norway***	***0.09***	Cyprus	10.2	***Norway***	***0.09***	Lithuania	18.3
Sweden	0.14	Greece	3.2	Sweden	0.14	Belgium	24.4	Sweden	0.14	France	10	Sweden	0.14	***UK***	***17***
***UK***	***0.32***	***Ireland***	**3.2**	***UK***	***0.32***	***Ireland***	***23.9***	***UK***	***0.32***	***UK***	***9.9***	***UK***	***0.32***	France	16.8
Slovenia	0.33	Belgium	3.1	***Slovenia***	***0.33***	***Netherlands***	***23.6***	Slovenia	0.33	Belgium	9.4	Slovenia	0.33	***Ireland***	***16.3***
Latvia	0.55	France	3.1	Latvia	0.55	Cyprus	23.2	Latvia	0.55	***Ireland***	***9.2***	Latvia	0.55	Slovakia	16.1
Iceland	0.58	Finland	3	Iceland	0.58	Finland	22.3	***Iceland***	***0.58***	***Denmark***	***8.8***	Iceland	0.58	***Norway***	***15.6***
Estonia	1.1	***Norway***	***2.9***	Estonia	1.1	Malta	21.4	Estonia	1.1	Luxembourg	8.7	Estonia	1.1	***Denmark***	***15.4***
***Ireland***	***1.18***	Spain	2.9	***Ireland***	***1.18***	***Norway***	***21.1***	***Ireland***	***1.18***	***Netherlands***	***8.6***	***Ireland***	***1.18***	Greece	*15.4*

Countries	J01M	Countries	*HL*	Countries	J01M	Countries	*NHL*	Countries	J01M	Countries	*MM*	Countries	J01M	Countries	*LEU*
***Norway***	***0.45***	Cyprus	4.6	***Norway***	***0.45***	Slovenia	*28.1*	***Norway***	***0.45***	***Iceland***	***11.7***	***Norway***	***0.45***	Belgium	21
***UK***	***0.45***	***UK***	***3.5***	***UK***	***0.45***	***UK***	***26.2***	***UK***	***0.45***	***Norway***	***11.3***	***UK***	***0.45***	Cyprus	18.9
Denmark	0.48	Italy	3.4	***Denmark***	***0.48***	***Denmark***	***25.1***	***Denmark***	***0.48***	Cyprus	10.2	***Denmark***	***0.48***	Lithuania	18.3
Sweden	0.68	Greece	3.2	Sweden	0.68	Belgium	*24.4*	Sweden	0.68	France	10	Sweden	0.68	***UK***	***17***
***Finland***	***0.76***	***Ireland***	***3.2***	***Finland***	***0.76***	***Ireland***	***23.9***	Finland	0.76	***UK***	***9.9***	Finland	0.76	France	16.8
Netherlands	0.77	Belgium	3.1	***Netherlands***	***0.77***	***Netherlands***	***23.6***	***Netherlands***	***0.77***	Belgium	9.4	Netherlands	0.77	***Ireland***	***16.3***
Estonia	0.83	France	3.1	Estonia	0.83	Cyprus	*23.2*	Estonia	0.83	***Ireland***	***9.2***	Estonia	0.83	Slovakia	16.1
***Ireland***	***0.83***	***Finland***	***3***	***Ireland***	***0.83***	***Finland***	***22.3***	***Ireland***	***0.83***	***Denmark***	***8.8***	***Ireland***	***0.83***	***Norway***	***15.6***
Iceland	0.91	***Norway***	***2.9***	Iceland	0.91	Malta	*21.4*	***Iceland***	***0.91***	Luxembourg	8.7	Iceland	0.91	***Denmark***	***15.4***
Czechia	0.95	Spain	2.9	Czechia	0.95	***Norway***	***21.1***	Czechia	0.95	***Netherlands***	***8.6***	Czechia	0.95	Greece	15.4

A higher overlap between the rank orders is possible indicating that the higher consumption of those antibiotics is associated with the higher incidence rate of hematological malignancies in the given countries. Similarly, the less consumption of the “inhibitory” antibiotics (J01D, J01M) seems to be associated with the higher incidence rate of hematological malignancies. Identical countries were written in *bold, italics, and underlined*. Concordance was considered, when six, out of the ten countries were identified in the rank order of hematological malignancies and antibiotic consumption.

## Results

The incidence of HL (estimated for 2020) showed strong positive association with the consumption of tetracycline (J01A) according to Spearman calculation ***(r* = *0.399, p* = *0.029***). A similar tendency for positive correlation was observed between HL incidence and the total consumption (J01) of antibiotics for systemic use *(****r* = *0.321, p* = *0.084****).* Positive significance was found between the consumption of narrow spectrum, beta-lactamase resistant penicillin (J01CF), and the incidence of NHL (***r* = *0.580, p* = *0.001***), while a strong negative association was found between cephalosporin consumption (J01D) with the incidence of NHL (***r* = **− ***0.460, p* = *0.011***) and the quinolone (J01M) with NHL (***r* = *0.380, p* = *0,038***) indicating the possible enhancing effect of tetracycline and the inhibitory effect of cephalosporin and quinolone in the development of NHL.

The incidence of MM demonstrated positive associations with the consumption of tetracycline (J01A) (***r* = *0.492, p* = *0.006***), with penicillin (J01C) *(****r* = *0.366, p* = *0.047***), and narrow spectrum, beta-lactamase resistant penicillin (J01CF), (***r* = *0.574, p* = *0.001***). Tendency of negative correlation appeared with cephalosporin, similarly to NHL (***r* = *− 0.34, p* = *0.063***). Multivariate ordinal logistic regression (OR) indicated increased risk between HL and the total consumption of systemic antibiotics (J01 p: 0.038), and tetracycline (J01A p: 0.002). Similarly, increased risk has been detected between the MM and tetracycline (J01A p: 0.002), and narrow spectrum, beta-lactamase resistant penicillin (J01CF p: 0.042) and decreased risk between cephalosporin and MM (J01D p:0.022). LEU showed increased risk with the consumption of macrolides (p: 0.047).

Comparing rank orders (first ten positions) of different hematological malignancies with the highest consumption rank order of "enhancer" antibiotics (J01A, J01C, J01CF), we have identified six countries identical with the rank order of HL, MM, and J01A (tetracycline). Six countries were identified in the NHL group and J01CF (narrow spectrum, beta-lactamase resistant penicillin), and seven countries were identified in the MM rank order with the J01CF class of antibiotics. Seven countries, out of ten, were identical with the HL rank order and the highest consumption of the penicillin group (J01C) and six with the NHL rank order. This concordance supports the possible associations between the consumption of different antibiotic classes and certain hematological malignancies.

Similarly, the lowest consumption of "inhibitor" antibiotics is in concordance with the higher incidence of different hematological malignancies. Six countries with the lowest consumption of cephalosporin (J01D) group of antibiotics are identical with the highest incidence (first ten positions) of the rank order of NHL and six are identical with the consumption of quinolone and the NHL.

## Discussion

The most densely populated microbial ecosystem that colonizes the human body is found in the gut and is commonly referred to as gut microbiota. It might be considered that gut microbiota is a separate organ itself, and the latest study sets an estimation of over 40 trillion intestinal microorganisms, bringing the ratio closer to 1:1 to somatic cells, expected to be around 30 trillion. The bacteria that comprise the mammal gut microbiota belong primarily to four phyla: *Firmicutes, Bacteroidetes, Proteobacteria,* and *Actinobacteria*. Altogether, these phyla account for over 95% of the total bacteria in the mammalian microbiota. The mean total mucosal surface of the digestive tract can be estimated as @32 m^2^, of which about 2 m^2^ refers to the large intestine^34^. The colonization of the intestinal lumen begins at birth and the composition of the gut microbiota is being influenced by several host and external factors and plays a crucial role in maintaining intestinal homeostasis, plays role in the maturation and education of the human immune system, protecting against the colonization of pathogen bacteria, responsible for energy harvest, production of nutrients and vitamins, metabolism of xenobiotics and procarcinogens. One of the most important scientific discoveries of recent years was the disclosure that the intestinal microflora takes part in the bidirectional communication between the gut and the brain. Scientists suggest that human gut microflora may even act as the "second brain"^35^.

Advances in culture-independent research techniques have led to an increased understanding of gut microbiota and the role it plays in health and disease. Several studies indicate the implication of altered microbiome (dysbiosis) in different metabolic disorders (diabetes, obesity)^36–38^, inflammatory bowel disease^39–41^ autism^42–44^, and neurodegenerative diseases, like Parkinson’s disease^45–48^, Alzheimer disease^49,50^ multiple scleroses^51–53^. Recent scientific advances have significantly contributed to our understanding of the complex connection between the microbiome and cancer, solid tumors, and hematological malignancies alike^6,54–56^. As it appears in the literature, microorganisms and microbial elements such as lipopolysaccharides (LPS) can up-regulate Toll-like Receptors (TLR)s, which can provoke activation of nuclear factor-kB (NF-kB), which is critical for controlling tumor-associated inflammation^57,58^, invasion, growth, survival, and immunosuppression^59^. Bacterial lipopolysaccharide (LPS) has also been demonstrated to hasten cell proliferation by c-Jun N-terminal Kinase activation^60^. According to reports, different hematological malignancies might show some association between the alterations of gut flora. It was possible to differentiate between the leukemia subjects and the controls based on their microbiota composition. The principal taxa comprise Roseburia, Ruminococcus, Anaerostipes, and Coprococcus with moderately higher abundance in the controls^61^. In a small „twin studies” difference was identified between the microbiome of the survivors of HL, and their unaffected co-twin controls, as it appears to have a deficit of rare gut microbes^62^. The microbiota affects hematopoiesis and influences the efficacies of chemotherapy and antimicrobial treatments^63^. The most extensively used gut microbial flora disruptors are antibiotics, and hence, it can be easily suspected that antibiotics might play a role in producing dysbiosis and contributing to the development of consecutive, non-infectious diseases. On talking about antibiotics producing dysbiosis, we must assume that different classes of antibiotics with different actions on certain microbial taxes might be diverse, as antibiotic sensitivity of gut flora microbiomes is different^64^.

## Conclusion

Scientific publications, cited in the References, clearly describe the role of the microbiome in the development of different hematological malignancies. Our study raises the possibility that different antibiotics, by influencing the composition of gut flora (microbiome), might influence the oncogenic process through the gut- brain axis, and through other molecular pathways, which might enhance or inhibit the development of different hematological malignancies. Tetracycline /J01A/, penicillin /J01C/ and particularly narrow spectrum, beta- lactamase resistant penicillin /J01CF/ appears to be promoting the development of certain hematological malignancies (HL, NHL, MM), while other groups of antibiotics might inhibit the oncogenic process (cephalosporin, J01D) through the modification of gut flora. The higher consumption rate of “enhancer” and the lower consumption of “inhibitor” antibiotics appears to be associated with the higher incidence of hematological malignancies as it is featured in the comparison of the rank order of hematological malignancies and antibiotic consumption (Table 2).

We did not find associations between LEU and the consumption of any major classes of antibiotics with Spearman correlation, which might be attributed to the heterogeneity of the leukemia group (myeloid, lymphoid, acute, chronic, etc.), but OR indicated a higher risk with macrolide consumption (p: 0.047). It is suspected that different subclasses of LEU might exhibit opposing effects when compared to antibiotic consumption, and hence, it would not appear when leukemia subgroups are compared together with the antibiotics when applying Spearman method.

### Strength of the paper

Comparing large, publicly available databases of the incidence of hematological malignancies (HL, NHL, MM, LEU) and the average yearly antibiotic consumptions (2009–2019) published in the ECDC yearly reports from 30 European Countries, indicates the possible role of certain antibiotic classes (tetracycline /J01A/, penicillin /J01C/, narrow spectrum, beta-lactamase resistant penicillin /J01CF/) in the development of some hematological malignancies (HL, NHL, MM, LEU). Cephalosporin consumption appears to reduce the risk of those malignancies. This effect can appear through the modification of gut flora.

### Weaknesses

This survey could not demonstrate the above association at the individual level, showing the direct effect of antibiotics and the development of hematological malignancies. As an ecological study, the results are basically suitable for generating a hypothesis. To rule out errors conclusions can be drawn only with strong constraints. As research to explore a possible correlation, this provides evidence that there may be correlations that could be examined in the future by analyzing data collected at the individual level.

## Abbreviations

ECDC: European Centre for disease prevention and control
SDI: Sociodemographic Index
HMs: Hematological malignancies
HL: Hodgkin lymphoma
NHL: Non-Hodgkin lymphoma
MM: Multiple myeloma
LEU: Leukemia
ECIS: European Cancer Information System
